# Multiresidue Pesticide Analysis in Tea Using GC–MS/MS to Determine 12 Pesticide Residues (GB 2763-2021)

**DOI:** 10.3390/molecules27238419

**Published:** 2022-12-01

**Authors:** Kunming Zheng, Rongmei Lin, Xuezhi Liu, Xiaoping Wu, Rongfeng Chen, Mengquan Yang

**Affiliations:** 1 Fujian CCIC-Fairreach Food Safety Testing Co., Ltd., Fuzhou 350001, China; 2 College of Plant Protection, Henan Agricultural University, Zhengzhou 450002, China; 3 Chinese Academy of Inspection and Quarantine Comprehensive Test Center, Beijing 100123, China; 4 National Center for Occupational Safety and Health, NHC, Beijing 102308, China; 5 College of Tobacco Science, Henan Agricultural University, Zhengzhou 450002, China; 6 Graduate School of Pharmaceutical Sciences, The University of Tokyo, Bunkyo-ku, Tokyo 113-8654, Japan

**Keywords:** gas chromatography–tandem mass spectrometry, solid-phase extraction, pesticide residues, multiple reaction monitoring, GB 2763-2021

## Abstract

Pesticides are widely used on tea plants, and pesticide residues are of significant concern to consumers. The National Food Safety Standard Maximum Residue Limits for Pesticides in Food (GB 2763-2021) was recently amended. However, detection methods for pesticides newly added to the list of residues in beverages have not yet been established. For that reason, this study developed a solid-phase extraction (SPE) and gas chromatography–tandem mass spectrometry (GC–MS/MS) method for determining the residues of 12 pesticides, including four newly added, in black and green tea. Sample preparation processes (sample extraction, SPE clean-up, elution solvent, and elution volume) were optimized to monitor these residues reliably. Multiple reaction monitoring (MRM) was used for GC–MS/MS electron impact (EI) mode determination. Finally, satisfactory recoveries (70.7–113.0% for green tea and 72.0–99.1% for black tea) were achieved at three concentrations (10 μg/kg, 20 μg/kg, and 100 μg/kg). The LOQs were 0.04–8.69 μg/kg, and the LODs were 0.01–3.14 μg/kg. This study provides a reliable and sensitive workflow for determining 12 pesticide residues in tea, filling a gap in the newly revised National Standards.

## 1. Introduction

Among three common non-alcoholic beverages (coffee, cocoa, and tea), tea is the most popular worldwide due to its high content of polyphenols, caffeine, and aromatic substances, which have anticancer, antioxidant, and anti-inflammatory properties [1,2]. In addition, as a widely consumed beverage, tea has historically provided significant economic benefits. To ensure the quality of tea, a large number of pesticides are used during cultivation, drying, and processing; consequently, tea leaves contain more pesticide residues than other crops. Massive pesticide exposure can lead to pesticide accumulation in the body, which harms human health and causes various diseases. To reduce the harmful effects of pesticide residues in humans, China has continuously revised the maximum residue standards for tea, expanding the pesticide range to 106 pesticide residue limits.

**Figure 1:**
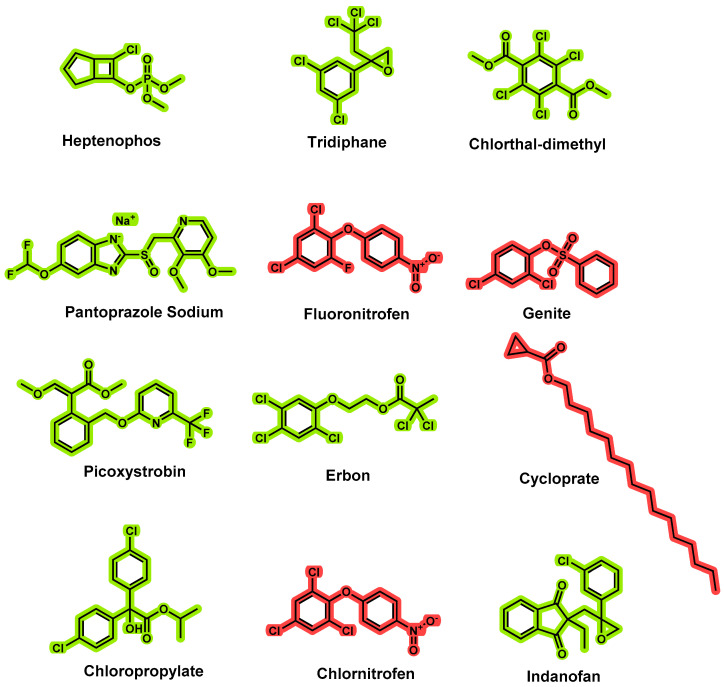
Structures of 12 pesticides (4 newly added, red) for residue determination.

To detect pesticide residues in tea, liquid chromatography–tandem mass spectrometry (LC–MS/MS) and gas chromatography–tandem mass spectrometry (GC–MS/MS) with MRM detection mode have been widely utilized [3,4,5,6,7]. To improve the precision of experimental results, pretreatment purification methods are required due to the presence of hundreds of chemical substances in tea that can cause significant interference during the detection process. Currently, QuEChERS [8,9,10] and solid-phase extraction (SPE) account for the majority of the pretreatment of tea [11,12]. Although QuEChERS is simple and quick to operate, its clean-up effect is significantly inferior to solid-phase extraction, particularly when the matrix is tea. Moreover, solid-phase extraction has a very high recovery rate and can reduce instrument maintenance costs. Residue detection methods for the simultaneous detection of 12 pesticides in tea (heptenophos, tridiphane, chlorthal-dimethyl, pantoprazole sodium, fluoronitrofen, genite, picoxystrobin, erbon, cycloprate, chloropropylate, chlornitrofen, and indanofan, Figure 1) have not been reported or determined using GC–MS/MS until now.

This study established and optimized a workflow for determining 12 pesticide residues by combining SPE clean-up and GC–MS/MS. This workflow satisfies the requirements for detecting multiple pesticide residues in tea and provides technical support for establishing standards for newly added pesticide residues.

## 2. Results and Discussion

### 2.1. Optimization of GC–MS/MS Condition

**Figure 2:**
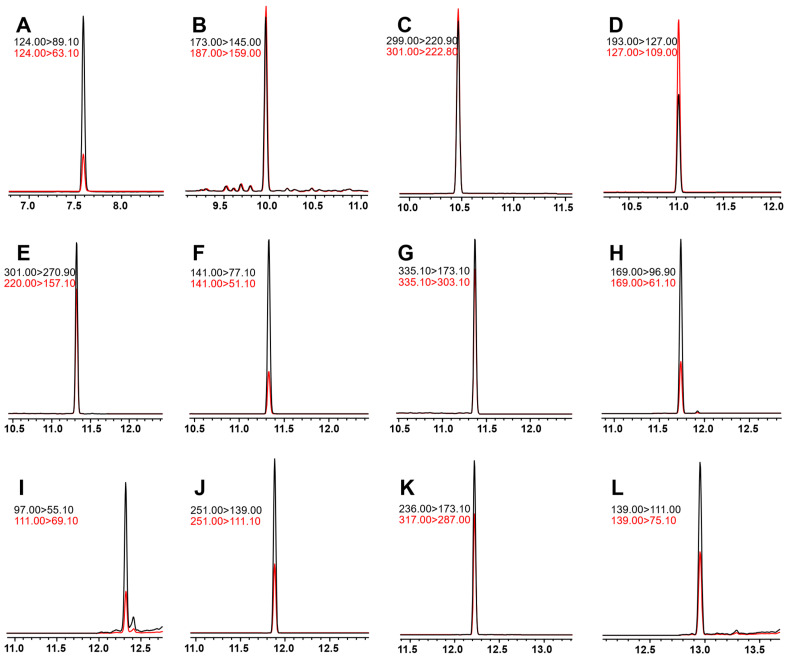
Extracted ion chromatogram of 12 standard pesticide solutions. (**A**) heptenophos, (**B**) tridiphane, (**C**) chlorthal-dimethyl, (**D**) pantoprazole sodium, (**E**) fluoronitrofen, (**F**) genite, (**G**) picoxystrobin, (**H**) erbon, (**I**) cycloprate, (**J**) chloropropylate, (**K**) chlornitrofen, (**L**) indanofan.

All tested pesticides’ GC and MS parameters were optimized using GCMS Solution version 4.45 and Microsoft Excel^TM^-based files (MRM Optimization Tool and GC/MS/MS pesticide database version 1.01). Before S-parameter optimization, all compounds were analyzed in full scan mode between 45 and 450 m/z. MS was subsequently operated in MRM mode. Using the Shimadzu SMART MRM Optimization Tool, the three most intense transitions and optimal collision energies (CE) were determined for each pesticide. Table 1 summarizes the operational parameters of the MS, including MRM transitions, retention times, CE, and dwell times. In addition, detector voltage was optimized absolute and relative to the tuning result modes (up to 0.5 kV). Regarding chromatographic peak heights, a detector voltage of 2 kV resulted in the best sensitivity. Mixtures of the 12 pesticides’ standards were detected using the optimized method, and the extracted ion chromatograms (EIC) are shown in Figure 2.

### 2.2. Optimization of Sample Preparation

#### 2.2.1. Optimization of Extraction Method

**Figure 3:**
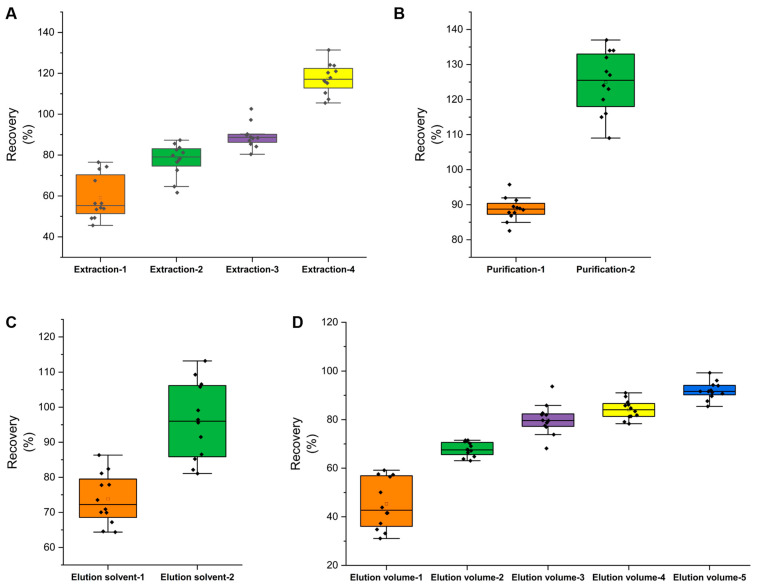
Optimization of sample preparation procedures: (**A**) extraction methods, (**B**) SPE columns, (**C**) elution solvents, (**D**) elution volumes.

**Figure 4:**
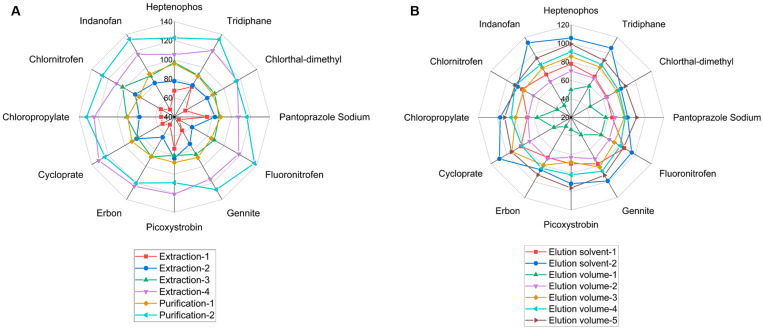
Recoveries of pesticides based on optimization of sample preparation. (**A**) Extraction methods and purification methods, (**B**) Elution solvents and elution volumes.

According to EN15662-2018, extraction of the target can be enhanced by soaking it in water when the sample’s water content is below 10% [13]. ACN is typically used to extract pesticide traces because it reduces co-extractions and improves extraction efficiency [8]. The effects of the following four extraction techniques were studied: extraction-1: direct extraction with 20 mL of ACN; extraction-2: extraction with 5 mL of water followed by 20 mL of ACN; extraction-3: extraction with 10 mL of water followed by 20 mL of ACN; extraction-4: extraction with 15 mL of water followed by 20 mL of ACN. The recoveries of the 12 pesticides were investigated by adding a mixed standard solution (0.02 mg/kg) to the tea samples. The results revealed that the pesticide recoveries were 45.6–76.5% (extraction-1), 61.6–87.3% (extraction-2), 80.4–102.6% (extraction-3), and 105.5–134.15% (extraction-4) (Figure 3A and Figure 4A). It was demonstrated that soaking in water prior to ACN extraction significantly improved extraction efficiency. Extraction-3 can be used in future experiments.

#### 2.2.2. Selection of Solid-Phase Extraction (SPE)

The experiment examined the effects of four commonly used Cleanert SPEs for tea: Florisil, Carb/NH_2_, Carb/PSA, and TPT. The results indicated that Florisil and Carb/NH_2_ were not as effective as Carb/PSA and TPT SPE (the fraction after Florisil and Carb/NH_2_ was dark in color); consequently, Carb/PSA and TPT SPE were chosen for further research. According to Figure 3B, the recoveries of the Carb/PSA solid-phase extraction ranged between 109.1% and 137.2%, and the recoveries of the Cleanert TPT SPE ranged between 82.5% and 95.7% (Figure 3B and Figure 4A). The Cleanert Carb/PSA SPE results revealed a severe matrix effect; consequently, Cleanert TPT SPE was deemed appropriate for future experiments.

Based on the results, we hypothesized that the loading materials of Carb/PSA SPE and TPT SPE produced disparities. Cleanert Carb/PSA SPE is loaded with two materials (graphitized carbon black and ethylenediamine-N-propyl). In contrast, Cleanert TPT SPE is a composite of three materials (graphitized carbon black, amine-modified silica, and amide-modified polystyrene), with the amine-modified silica and amide-modified polystyrene in the column being quite effective on alkaloids.

#### 2.2.3. Eluent Conditions

According to the polarities of the 12 pesticides and the properties of the Cleanert TPT SPE, the elution solvents for spiked recovery of the 12 pesticides were determined to be ACN–toluene (3:1) and acetone–hexane (1:1). According to the results of the experiment, the capacities of these two solvents to elute the 12 pesticides were comparable (Figure 3C and Figure 4B). Given that toluene is a strictly regulated and more toxic chemical, acetone–hexane (1:1) was chosen as the elution solvent for further research. To determine the effects of elution volume, the elution solvent was supplemented with 5, 10, 15, 20, and 25 mL at a rate of 5 mL per time. After the fractions were concentrated, they were subjected to GC–MS/MS analysis. The recoveries of the 12 pesticides were more than 85% when the eluent volume was 25 mL (Figure 3D and Figure 4B). Therefore, the final elution volume was determined to be 25 mL.

#### 2.2.4. Combination of the Optimized Procedures

The results of single-factor optimization (extraction methods, purification methods, elution solvents, and elution volumes) are depicted in Figure 4. It was demonstrated that extraction-4 and purification-1 (Figure 4A) and elution solvent-2 and elution volume-5 (Figure 4B) were the methods that achieved the greatest recoveries. Since elution solvent-2 (ACN–toluene (3:1)) contains toluene, which is toxic and strictly controlled, elution solvent-2 (acetone–hexane (1:1)) was chosen for further research.

### 2.3. Method Evaluation

#### 2.3.1. Linear Range, Limit of Detection (LOD), and Limit of Quantification (LOQ)

In accordance with the optimized experimental conditions, a series of standard solutions containing concentrations of 0, 10, 20, 50, 100, 200, 500, and 1000 μg/L were prepared from the dilutions of the 12 pesticide standard solutions with blank tea sample extracts to calculate the standard curve. With a three-fold signal-to-noise ratio, the limits of detection (LODs) and limits of quantification (LOQs) of the 12 pesticides were calculated. The results indicated that the correlation coefficients between the concentrations of the 12 pesticide compounds and their peak areas in the range of 10–1000 μg/L were all greater than 0.9976.

The limits of detection (LODs) and limits of quantification (LOQs) of the 12 pesticides in green tea were 0.01–3.14 μg/kg and 0.04–8.69 μg/kg, respectively. As indicated in China’s National Food Safety Standard Maximum Residue Limits for Pesticides in Food (GB 2763-2021) (http://down.foodmate.net/standard/sort/3/97819.html, accessed on 3 March 2021), the LOQs for all 12 pesticides were significantly lower than the maximum residue levels specified, indicating that the method described in this paper meets the actual detection requirements. Finally, satisfactory recoveries (70.7–113.0% for green tea and 72.0–99.1% for black tea) were achieved at three concentrations (10 μg/kg, 20 μg/kg, and 100 μg/kg).

#### 2.3.2. Matrix Effect

The matrix effect refers to the presence of substances other than the target that appear to inhibit or boost the detection signal of the standard solution of the pure solvent. Tea contains pigments, caffeine, minerals, and other substances that reduce the influence of endogenous substances on the precision of test results. The matrix effect was determined using the slope ratio of the matrix standard curve to the solvent standard curve [14]. Less than 0.9 indicated a matrix inhibition effect, while greater than 1.1 indicated a matrix enhancement effect [15]. According to the experimental results, the ratios of the 12 pesticides were 1.21–1.48, indicating that the 12 pesticides exhibited matrix effects; therefore, a matrix-matched standard curve was utilized for quantification.

#### 2.3.3. Spiked Recovery and Precision

Three mixed standard solutions of 10 μg/kg, 20 μg/kg, and 100 μg/kg were added to the blank tea matrix, and six parallel experiments were performed for each spiked level. The conditions were optimized for the determination. Based on the optimized workflow for these pesticides, black and green tea from the market in Fujian Province, China (black tea from Wuyi Mout. Zichen Tea Industry Co., Ltd.; green tea from Shiyi Tongyuan Biotechnology Co., Ltd.), were utilized to determine the pesticide residues in tea. As shown in Table 2 and Table 3, this workflow can effectively determine these 12 pesticide residues. Thus, it is evident that the method offers excellent accuracy and precision and can be used to determine these 12 pesticide residues in tea samples [16].

### 2.4. Pesticide Residue Determination in Different Tea Samples

Based on the optimized workflow, 20 tea samples available for sale were used for pesticide residue determination. Picoxystrobin was detected in 6 of 20 (30%) samples, with a concentration ranging from 0.04 to 0.15 mg/kg, which is much lower than the limitation (20 mg/kg). The other 11 pesticides were not detected in the 20 tea samples (Table 4). It was determined that these 12 pesticide residues were below the limitation; therefore, the tea we tested is safe for human consumption.

## 3. Materials and Methods

### 3.1. Chemicals and Materials

BePure supplied the pesticides (heptenophos, tridiphane, chlorthal-dimethyl, pantoprazole sodium, fluoronitrofen, genite, picoxystrobin, erbon, cycloprate, chloropropylate, chlornitrofen, and indanofan). Anhydrous sodium sulfate (Beijing Chemical Factory, Beijing, China) was cauterized at 550 °C for 4 h, placed in a desiccator, and cooled to make it ready for use. Agela Technologies supplied Fosrisil (500 mg, 6 mL), Carb/NH_2_ (500 mg, 6 mL), Cleanert TPT (2 g, 6 mL), and Carb/PSA (500 mg, 6 mL) SPEs. All chemicals and solvents utilized in this investigation were of analytical purity. Triple-distilled water was used in this study. The black and green tea utilized in this work were acquired from the Fuzhou market.

### 3.2. Standard Solutions and Calibration Curves

The 100 mg/L working standard solutions of the 12 pesticides were dissolved in acetone and stored at −20 °C. The mixed standard solutions were prepared in acetone at a concentration of 2 mg/L and stored at −20 °C. To calculate the calibration curves, working standard solutions were prepared by diluting the mixed standard solutions in a concentration series of 0.0 μg/L, 20 μg/L, 50 μg/L, 100 μg/L, 200 μg/L, 400 μg/L, 800 μg/L, and 1000 μg/L.

### 3.3. Extraction and Clean-Up

The tea was ground and passed through a 20-mesh sieve, and a 5.0 g (accurate to 0.01 g) sample of the ground tea was weighed. Next, the samples were soaked in varying volumes of water for 30 min: 0 mL (extraction-1), 5 mL (extraction-2), 10 mL (extraction-3), and 15 mL (extraction-4). After adding 20 mL of acetonitrile (ACN), the mixture was vigorously vortexed for 5 min. The samples were then centrifuged at 4000 rpm/min for 5 min. Then, 10 mL of the acetonitrile layer was drawn into a new centrifuge tube for purification.

For clean-up, 4 mL of supernatant was passed through either a Cleanert TPT (purification-1) or Carb/PSA (purification-2) SPE with approximately 2 cm of anhydrous sodium sulfate. Two solvents for elution were tested for this step: (1) For column activation, 25 mL of elution solvent-1 (acetone–hexane (1:1)) was used; for elution, a specific volume of acetone–hexane (1:1) was used. (2) 25 mL of elution solvent-2 (ACN–toluene (1:3)) was used for column activation. For elution, a specific volume of ACN–toluene (1:3) was used. For elution volumes, 5 mL (elution volume-1), 10 mL (elution volume-2), 15 mL (elution volume-3), 20 mL (elution volume-4), and 25 mL (elution volume-5) were tested.

The gathered fraction was concentrated using a rotary evaporator. For GC–MS/MS analysis, the mixtures were diluted into 1 mL of corresponding solvent and passed through a 0.22 m filter membrane.

### 3.4. GC–MS/MS Analysis

Pesticide concentrations were determined using tandem mass spectrometry (GCMS-TQ 8040, Shimazu Corp., Japan). GC separation was performed using an Agilent HP-5 MS (30 m × 0.25 mm × 0.25 μm) capillary column. The following conditions were set for the oven: 50 °C, maintain for 1 min; 50–150 °C at intervals of 25 °C/min, hold for 1 min; 150–300 °C at intervals of 10 °C/min, hold for 5 min. The temperature at the inlet was set to 250 °C. With a 2.0 mL/min flow rate, 99.999% pure helium was used as the carrier gas for chromatographic analysis. An injection volume of 2 μL was analyzed in the splitless mode under high pressure conditions (200 kPa).

A triple quadrupole mass spectrometer in electron impact (EI) ionization mode was operated with a 70 eV ionization voltage and 60 µA of emission current. The interface (transfer line to the tandem MS), ion source, and quadrupole temperatures were maintained at 230 °C and 150 °C. Multiple reaction monitoring (MRM) mode was used for target detection.

### 3.5. Method Validation

For each pesticide, the precision, accuracy, linearity, limit of detection (LOD), and limit of quantification (LOQ) were calculated following European Commission guidelines to validate the analytical method used in this study [16]. Working solutions containing pesticide concentrations ranging from 10.0 to 1000.0 µg/L were prepared and utilized to generate calibration curves. Each working solution was analyzed three times, and the peak area ratios of each pesticide standard to internal standard were determined. Minimum analyte concentrations in spiked blank samples inducing MRM traces with signal-to-noise ratios (S/N) of 3 and 10, respectively, were used to calculate the LOD and LOQ values. Recovery of the analytical method was evaluated by adding the standard pesticide mixture to the sample matrix at three different concentrations (10.0, 20.0, and 100.0 µg/kg) and then extracting and analyzing these pesticides according to the previously described method.

### 3.6. Statistical Analysis

The collected data were analyzed using Origin (version 2021) software.

### 3.7. Application of the Optimized Workflow for Pesticide Residues

Twenty tea samples available for sale were purchased for pesticide residue determination. An amount of 200 g was ground into powder for each sample, and 5 g of each was used for further determination (following optimized procedures).

## 4. Conclusions

This study optimized the sample preparation workflow (extraction methods, SPE columns, elution solvents, and elution volume) for 12 pesticides in GB 2763-2021. A combination of SPE and GC–MS/MS was used to simultaneously determine the residues of 12 pesticides in tea. The method demonstrated simple operation, rapidity, and high qualitative and quantitative accuracy. It is applicable for the detection of 12 pesticide residues in tea. The optimized workflow provides technical support for food safety and fills the gap for newly added pesticide residues, particularly in tea.

## Figures and Tables

**Table 1:** GC–MS/MS detection conditions for 12 pesticides.

Pesticide	Retention Times/min	Dwell Times/ms	Quantitative Mass/ m/z	Collision Energy/V	Qualitative Ion Pair, m/z	Collision Energy/V
Heptenophos	7.567	25	124.00 > 89.10	9	124.00 > 63.10	27
Tridiphane	9.879	25	173.00 > 145.00	15	187.00 > 159.00	12
Chlorthal-dimethyl	10.475	25	299.00 > 220.90	24	301.00 > 222.80	27
Pantoprazole Sodium	11.065	25	193.00 > 127.00	5	127.00 > 109.00	15
Fluoronitrofen	11.453	25	301.00 > 270.90	12	220.00 > 157.10	27
Genite	11.345	25	141.00 > 77.10	6	141.00 > 51.10	27
Picoxystrobin	11.377	25	335.10 > 173.10	9	335.10 > 303.10	6
Erbon	11.745	25	169.00 > 96.90	15	169.00 > 61.10	30
Cycloprate	12.319	25	97.00 > 55.10	10	111.00 > 69.10	8
Chloropropylate	11.876	25	251.00 > 139.00	14	251.00 > 111.10	26
Chlornitrofen	12.243	25	236.00 > 173.00	24	317.00 > 287.00	12
Indanofan	12.957	25	139.00 > 111.00	15	139.00 > 75.10	27

**Table 2:** Linear equations, limits of detection, and limits of quantification, precision of 12 pesticides for green tea.

Pesticide	Green Tea				Spiked Level					
					10 μg/kg		20 μg/kg		100 μg/kg	
	Linear Equation	R^2^	LOD (ug/kg)	LOQ (ug/kg)	Recovery (%)	RSD (%)	Recovery (%)	RSD (%)	Recovery (%)	RSD (%)
Heptenophos	f(x) = 1502.694572 ∗ x − 113.0040	0.9994	0.01	0.04	101.6	6.6	96.0	7.7	103.6	3.2
Tridiphane	f(x) = 560.965491 ∗ x − 45.0606	0.9994	1.49	4.97	102.2	8.9	91.4	9.9	93.9	3.9
Chlorthal-dimethyl	f(x) = 383.149211 ∗ x − 24.5600	0.9997	0.06	0.21	86.5	11.6	87.0	10.6	90.5	3.5
Pantoprazole Sodium	f(x) = 818.174622 ∗ x − 78.9870	0.9998	0.02	0.07	83.2	10.5	86.3	8.0	90.6	2.0
Fluoronitrofen	f(x) = 365.018769 ∗ x − 65.8542	0.9989	0.06	0.21	70.7	7.7	86.3	9.2	91.4	1.3
Genite	f(x) = 2082.333399 ∗ x − 145.0708	0.9997	0.01	0.04	95.1	8.6	87.9	8.9	94.2	2.7
Picoxystrobin	f(x) = 140.532755 ∗ x − 3.5060	0.9999	0.13	0.43	79.5	10.9	81.5	10.9	89.3	4.3
Erbon	f(x) = 938.903891 ∗ x − 21.0300	0.9996	0.11	0.38	94.8	8.8	90.2	8.3	94.3	3.1
Cycloprate	f(x) = 2157.633788 ∗ x − 231.0450	0.9983	2.61	8.69	109.4	6.1	90.8	11.5	101.6	4.9
Chloropropylate	f(x) = 1647.330772 ∗ x − 121.0034	0.9999	0.11	0.35	88.1	6.5	88.8	8.9	92.7	2.8
Chlornitrofen	f(x) = 272.771764 ∗ x − 12.0450	0.9999	0.08	0.27	76.4	10.7	80.4	7.6	90.3	2.9
Indanofan	f(x) = 632.674102 ∗ x − 32.4315	0.9998	0.31	1.04	113.0	2.9	93.6	7.0	93.7	3.2

**Table 3:** Linear equations, limits of detection, and limits of quantification, precision of 12 pesticides for black tea.

Pesticide	Black Tea				Spiked Level					
					10 μg/kg		20 μg/kg		100 μg/kg	
	Linear Equation	R^2^	LOD (ug/kg)	LOQ (ug/kg)	Recovery (%)	RSD (%)	Recovery (%)	RSD (%)	Recovery (%)	RSD (%)
Heptenophos	f(x) = 1079.505741 ∗ x − 864.5287	0.9993	0.01	0.08	97.2	7.8	93.2	3.8	94.9	6.0
Tridiphane	f(x) = 396.966791 ∗ x − 561.8339	0.9994	1.76	6.57	99.1	12.7	87.9	3.3	89.0	5.8
Chlorthal-dimethyl	f(x) = 257.457728 ∗ x − 125.6542	0.9996	0.06	0.24	74.2	14.2	84.9	5.9	87.2	4.6
Pantoprazole Sodium	f(x) = 576.950434 ∗ x − 674.7645	0.9996	0.03	0.08	77.2	8.7	80.6	5.5	85.8	6.1
Fluoronitrofen	f(x) = 247.444329 ∗ x − 127.4367	0.9996	0.05	0.30	72.0	13.8	80.3	6.5	85.8	4.5
Genite	f(x) = 1518.161305 ∗ x − 6453.9763	0.9976	0.01	0.03	90.8	10.8	87.5	2.3	88.9	6.5
Picoxystrobin	f(x) = 95.176043 ∗ x − 125.5432	0.9978	0.24	0.44	78.7	9.9	75.7	7.2	84.7	5.2
Erbon	f(x) = 658.202230 ∗ x − 786.5413	0.9997	0.44	0.3	84.9	8.5	84.7	3.0	88.1	6.3
Cycloprate	f(x) = 1682.980785 ∗ x − 154.3278	0.9996	3.14	7.37	98.8	13.1	85.0	11.5	93.2	5.7
Chloropropylate	f(x) = 1129.119124 ∗ x − 125.4536	0.9994	0.13	0.37	91.7	13.0	86.9	3.5	88.6	7.0
Chlornitrofen	f(x) = 200.986221 ∗ x − 789.5482	0.9998	0.08	0.34	79.0	11.5	80.3	3.3	85.6	6.2
Indanofan	f(x) = 456.259727 ∗ x + 12.3457	0.9993	0.45	1.41	94.9	10.8	89.4	2.8	89.2	6.3

**Table 4:** Summary of the pesticide residues in 20 tea samples available for sale.

Pesticide	No. of Detection	Percent of Detection (%)	Range (mg/kg)	No. of Unqualified Samples	Percent of Unqualified Samples (%)	Limitation (mg/kg)
Heptenophos	0	0	/	/	/	0.01 *
Tridiphane	0	0	/	/	/	0.05 *
Chlorthal-dimethyl	0	0	/	/	/	0.01
Pantoprazole Sodium	0	0	/	/	/	0.05 *
Fluoronitrofen	0	0	/	/	/	0.01 *
Genite	0	0	/	/	/	0.01 *
Picoxystrobin	6	30%	0.04–0.15	0	0	20
Erbon	0	0	/	/	/	0.01 *
Cycloprate	0	0	/	/	/	0.01 *
Chloropropylate	0	0	/	/	/	0.02 *
Chlornitrofen	0	0	/	/	/	0.01 *
Indanofan	0	0	/	/	/	0.01 *

* Temporary limitation.

## Data Availability

The raw sequence data reported in this paper have been deposited in the Genome Sequence Archive at the BIG Data Center, Beijing Institute of Genomics (BIG), Chinese Academy of Sciences.
